# Role of Ubiquitination in PTEN Cellular Homeostasis and Its Implications in GB Drug Resistance

**DOI:** 10.3389/fonc.2020.01569

**Published:** 2020-09-02

**Authors:** Qin Xia, Sakhawat Ali, Liqun Liu, Yang Li, Xuefeng Liu, Lingqiang Zhang, Lei Dong

**Affiliations:** ^1^School of Life Sciences, Beijing Institute of Technology, Beijing, China; ^2^School of Electronic and Optical Engineering, Nanjing University of Science and Technology, Nanjing, China; ^3^State Key Laboratory of Proteomics, National Center for Protein Sciences, Beijing Institute of Lifeomics, Beijing, China

**Keywords:** glioma, glioblastoma, phosphatase and tensin homolog, ubiquitination, E3 ubiquitin ligases, drug resistance

## Abstract

Glioblastoma (GB) is the most common and aggressive brain malignancy, characterized by heterogeneity and drug resistance. PTEN, a crucial tumor suppressor, exhibits phosphatase-dependent (PI3K-AKT-mTOR pathway)/independent (nucleus stability) activities to maintain the homeostatic regulation of numerous physiological processes. Premature and absolute loss of PTEN activity usually tends to cellular senescence. However, monoallelic loss of PTEN is frequently observed at tumor inception, and absolute loss of PTEN activity also occurs at the late stage of gliomagenesis. Consequently, aberrant PTEN homeostasis, mainly regulated at the post-translational level, renders cells susceptible to tumorigenesis and drug resistance. Ubiquitination-mediated degradation or deregulated intracellular localization of PTEN hijacks cell growth rheostat control for neoplastic remodeling. Functional inactivation of PTEN mediated by the overexpression of ubiquitin ligases (E3s) renders GB cells adaptive to PTEN loss, which confers resistance to EGFR tyrosine kinase inhibitors and immunotherapies. In this review, we discuss how glioma cells develop oncogenic addiction to the E3s-PTEN axis, promoting their growth and proliferation. Antitumor strategies involving PTEN-targeting E3 ligase inhibitors can restore the tumor-suppressive environment. E3 inhibitors collectively reactivate PTEN and may represent next-generation treatment against deadly malignancies such as GB.

## Introduction

Glioblastoma (GB), a WHO grade IV glioma, is the most common and malignant adult brain tumor. GB comprises 54% of all gliomas and 45.2% of primary brain and central nervous system malignancies (1). Conventional treatments for GB include surgery, radiotherapy, and several pharmacological interventions that involve temozolomide (TMZ)-based chemotherapy and immunotherapy. Despite multimodal therapies, GB remains refractory because of genetic and epigenetic mutations, such as phosphatase and tensin homolog (PTEN), vascular endothelial growth factor receptor 2, epidermal growth factor receptor (EGFR), and cellular tumor antigen p53 mutations. These mutations induce the aberrant phosphoinositide 3-kinase (PI3K)/protein kinase B (AKT)/mammalian target of rapamycin (mTOR) signaling (2–4). GB, depending on molecular signatures, is divided into four subtypes, including classical, mesenchymal (MES), neural, and proneural (5). Among the subtypes, MES shows the most severe drug resistance and frequent mutations of PTEN (37%), accompanied by PI3K/AKT hyperactivation (6). Mutational studies suggest that the successive depletion of each PTEN allele is involved in the transition from low- to high- grade gliomas (7, 8). Notably, GB patients with PTEN deficiency have a considerably short median life expectancy and poor survival (9–12). Elucidating the mechanisms underlying tumorigenesis in GB mediated by PTEN loss is important and can reveal potential targets for treatment.

PTEN is a haplo-insufficient cancer repressor, and loss of PTEN function occurs in GB (13–15). Partial PTEN inactivation or a subtle decline in PTEN substantially induces susceptibility to cancer and tumorigenesis (16, 17), although its absolute loss stimulates cellular senescence (18–21). PTEN has phosphatase-dependent/independent activities in the cell and regulates numerous biological processes, including genomic stability, cell survival, proliferation, and metabolism. The lipid phosphatase-dependent activity of cytoplasmic PTEN dephosphorylates the phosphatidylinositol 3,4,5-triphosphate (PIP_3_) to phosphatidylinositol 4,5-biphosphate (PIP_2_) (22), thereby counteracting the PI3K-mediated AKT activation. This action modulates numerous downstream signaling events, including intense growth and proliferation, and abated apoptosis. PTEN also executes protein-phosphatase dependent roles such as maintenance of genomic stability in the nucleus (23). Moreover, PTEN as a scaffold protein in both the nucleus and the cytoplasm exerts part of its tumor-suppressive function independently of PIP_3_ and the PI3K/AKT axis.

Translocation and localization of PTEN in subcellular compartments (cytoplasm and nucleus) is very crucial, as it performs distinct homeostatic functions in each cell chamber. PTEN’s vital involvement in the homeostatic regulation of numerous cellular processes necessitates strict regulation of its expression. Transcriptional and post-transcriptional modulation of PTEN expression is mediated by multiple mechanisms, including epigenetically induced silencing, transcriptional repression, and regulation by miRNAs. Moreover, PTEN protein levels are managed by numerous post-translational modifications (PTMs), including phosphorylation, ubiquitination, and oxidation (24, 25). Ubiquitination efficiently regulates the PTEN protein level and fine-tunes PTEN nucleocytoplasmic transport. Numerous studies have emphasized the critical role of the ubiquitin-proteasome system in PTEN regulation, where NEDD4-1, XIAP, WWP2, and other E3s degrade PTEN via its polyubiquitination (26–28). Also, a PTEN deubiquitinating enzyme, ubiquitin C-terminal hydrolase 13 (USP13), can restore PTEN stability in tumors. This finding suggests that ubiquitination plays a crucial role in the homeostatic regulation of the antitumor features of PTEN.

This review discusses several topics, including the mechanism by which PTEN regulates a plethora of phenotypic features and numerous other regulatory mechanisms, how PTM-ubiquitination specifically modulates PTEN cellular expression, localization, and activity in GB, and the role of aberrant expression of E3 ligases in GB and their impact on PTEN activity. Given the crucial role in PTEN proteostasis, further understanding of E3s can help to identify novel prognostic markers and anticancer therapies.

## PTEN Structure and Activity

**FIGURE 1:**
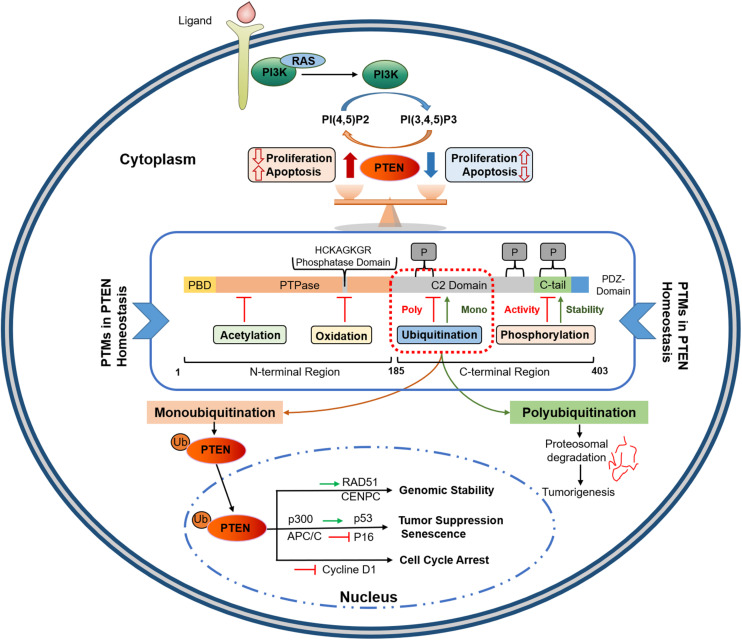
PTEN protein structure and its functional regulation by post-translational modifications. PTEN comprises 403 amino acids with five functional domains, including a PIP2-binding domain (PBD), a phosphatase domain having catalytic core, a C2 domain containing ubiquitination sites, two PEST domains for degradation, and a protein-protein interaction PDZ motif. Homeostatic balance of PTEN protein in cytoplasm regulates health proliferation and apoptosis in the cell. PTEN post-translational regulation involves ubiquitination within the PBD and C2 domains, oxidation and SUMOylation in the C2 domain, acetylation on PTPase and PDZ-binding sites, while phosphorylation occurs within the C2 domain and C-tail terminal. Polyubiquitination marks PTEN for Proteosomal degradation while Monoubiquitination transport it to nucleus. Nuclear PTEN performs several important functions, including genomic stability regulation via interacting with CENPC and RAD51, apoptosis induction by upregulating p53, and cell cycle arrest and senescence by inhibiting Cycline D1 and p16, respectively. PIP2, Phosphatidylinositol-4,5-bisphosphate; PEST, proline, glutamic acid, serine, threonine; PTPase, protein tyrosine phosphatase; PBD, PIP2-binding domain; APC/C, anaphase-promoting complex; CENPC, centromere protein C.

Phosphatase and tensin homolog gene, encoded by 9 exons and located on chromosome 10q23, has two distinct domains (tensin-like and a catalytic domain) and encodes 47 kDa dual-specificity protein phosphatase (403 amino acids) (29–32). As shown in Figure 1, PTEN protein consists of five functional domains, including a short N-terminal PIP_2_-binding domain (PBD), a catalytic phosphatase domain (i.e., protein tyrosine phosphatase, PTP), a lipophilic C2 domain or membrane-binding domain, a C-terminal tail, and a class I PDZ-binding (PDZ-BD) motif (33). PTEN protein functions in lipid/protein phosphatase-dependent and scaffold-dependent manner (23). The phosphatase domain (N-terminal) incorporates a consensus PI (4, 5) P2-binding (PIP_2_) motif; the lipophilic C2 domain of the PTEN C-terminal is involved in the accurate positioning of PTEN in the phospholipid plasma membrane. The C-terminal tail, including the last 50 amino acids, is crucial in protein stability owing to its several phosphorylation sites. The physiologic activity of PTEN is largely controlled by its N-terminal phosphatase domain; nearly 40% of tumorigenic mutations affect the C2 domain and the tail sequence. This suggests a crucial role of the C-terminal in maintaining PTEN function (34). Loss of lipid phosphatase activity is implicated in numerous PTEN loss-induced aberrant phenotypes. However, studies involving PTEN mutants (C124S: a double phosphatase mutant and G129E: a lipid phosphatase mutant) have shown that PTEN protein phosphatase activity is also essential for tumor suppression (35, 36). Many experimental systems comprising PTEN mutant forms, such as G129E and Y138L, have been used to characterize its protein phosphatase functions (23, 32); however, the physiological implications of protein dephosphorylation have yet to be elucidated. Therefore, both lipid and protein targets of PTEN should be considered separately to treat tumorigenesis induced by the loss of PTEN.

Molecular mechanisms that regulate dimerization also play important roles in PTEN function. The self-interaction of PTEN protein causes its dimerization and subsequent activation. PTEN homodimers are stabilized by its C-terminal tail, whose phosphorylation-induced closed conformation negatively affects PTEN homodimerization (37). Recent studies have indicated that homodimerized PTEN (PTEN-PTEN dimer) represents active conformation in which full phosphatase function can be exerted on its lipid substrate PIP_3_ (38). PTEN dimerization-mediated activation may be cell-context dependent, evidenced by PTEN protein extracted from insect cells is ubiquitously monomeric (39). Moreover, *in vitro* and *in vivo* studies have confirmed that mutant PTEN can form heterodimers with wild-type (wt) PTEN, inhibiting wt-PTEN catalytic activity (37).

Post-translational modifications, particularly phosphorylation and ubiquitination, can fine-tune the biochemical properties of PTEN. Upon phosphorylation, the C-terminal promotes PTEN conformational closure by facilitating interaction between its tail and C2 domain; C2 domain dephosphorylation results in PTEN open conformation which favors its binding to the membrane and the PDZ domain. Accordingly, phosphorylation of distinct serine and threonine residues (S380, T382, and T383) in the C-terminal tail modulates (activates/deactivates) the tail-dependent regulation of PTEN stability and activity (40). Phosphorylation-induced closed conformation inactivates PTEN in the cytoplasm (40, 41). These interactions can influence numerous PTEN properties, such as stability, subcellular partitioning, and binding affinity to the plasma membrane. Ubiquitination may be the most significant post-translational modification for PTEN, which is involved in PTEN transport to different subcellular locations and regulates its catalytic activity. For instance, the melanocyte-stimulating hormone receptor (MSHR or MC1R: melanocortin receptor 1) in melanocytes protects from ultraviolet-induced skin injury by preventing E3 ligase WWP2-mediated PTEN degradation (42). Therefore, extensive studies on the molecular mechanisms underlying the diverse interactions of PTEN in cells can identify novel strategies to restore PTEN function.

### Cytoplasmic Lipid Phosphatase-Dependent Function

Phosphatase and tensin homolog was first identified as a PTP given the close structural homology of its catalytic domain to the PTP family; however, it was subsequently revealed to mainly function as a lipid phosphatase (24, 32). PTEN dephosphorylates its major lipid substrate PIP_3_ to antagonize the activation of the proto-oncogenic PI3K-AKT-mTOR pathway that synchronizes various cellular processes such as proliferation and survival (43). The loss of PTEN lipid phosphatase activity, which hyperactivates the PI3K-AKT-mTOR pathway, promotes tumorigenesis (44, 45). PI3K-dependent signals help determine the regulation of several cellular homeostatic mechanisms; regardless, numerous settings exist, such as cell polarization in chemotaxis where localization of PIP_3_ at the subcellular level may be crucial (46–50). In such instances, PIP_3_ signal gradients seem established by the localized PTEN activity (51–54). Thus, PTEN-PI3K axis is a crucial functional axis that acts as a switch to regulate the status of numerous proto-oncogenic signals and is frequently deregulated during GB genesis (55).

### Nuclear PTEN Functions in a Phosphatase-Dependent/Independent Manner

Although its biological effects depend primarily on its ability to dephosphorylate lipid substrates, PTEN exhibits an inherent phosphatase activity for peptides phosphorylated on Tyr, Ser, and Thr. This attribute confers dual-specificity on PTEN as a protein phosphatase. PTEN causes the direct dephosphorylation of many protein substrates, and its auto-dephosphorylation has also been reported (56–59). PTEN physically associates with centromere protein-C to conserve chromosomal stability, which is a phosphatase-independent action, while it upregulates Rad51 to repair DNA double-strand breaks via phosphatase-independent activity. Nuclear PTEN performs numerous antitumor functions in other aspects, such as cell cycle arrest and senescence (Figure 1). Nuclear PTEN reduces cyclin D1, resulting in cell cycle arrest at G0/G1 (60). It also modulates cellular senescence by favoring the interaction between anaphase-promoting complex/cyclosome (APC/C) and cadherin-1 (CDH1) (61). The coordinated regulation of the C-terminal tail-dependent physical interactions of nuclear PTEN with p53 and microspherule protein 1 (MCRS1), and PTEN phosphatase activity could determine growth inhibition, cell cycle arrest, and apoptosis (62, 63).

Similarly, nuclear PTEN plays a crucial role in GB development. U87MG cells showed enhanced apoptotic DNA fragmentation attributed to nuclear PTEN overexpression (64). Moreover, in U251MG cells, nuclear PTEN facilitates G1 arrest and represses anchorage-independent growth (65). Ectopic expression of PTEN in GB cell lines harboring PTEN mutations (U87MG, U373MG, and U251MG) causes a p27-induced reduction in cyclin E/CDK2 activity and phosphorylation of retinoblastoma protein, leading to cell cycle arrest at the G-S phase (66). Nuclear PTEN functions mainly depend on its phosphorylation status. In one study, GB samples obtained from patients after TMZ and ionizing radiation (IR) treatment showed enhanced phosphorylation of nuclear PTEN. This PTEN phenotype was associated with the poor survival of the patients (67). Although total PTEN was higher in the cytoplasm, the amount of phosphorylated PTEN was higher in the nucleus. This difference suggests that mainly nuclear PTEN was phosphorylated after the treatment. IR treatment mainly damages DNA that can lead to cell death. Phosphorylation of nuclear PTEN on tyrosine 240 (pY240-PTEN) by FGFR2 enhances DNA repair, which facilitates GB growth. Thus, blocking Y240-PTEN phosphorylation improves radiation sensitivity to GB (68). In addition, nuclear PTEN expression is significantly downregulated and inversely associated with pyruvate dehydrogenase kinase 1 expression in various brain metastases (69). These studies emphasize the significance of nuclear PTEN and suggest that the deregulation of nuclear PTEN phosphorylation could be implicated in gliomagenesis.

### PTEN Subcellular Translocation

Phosphatase and tensin homolog translocation between the cytoplasm and the nucleus, as well as subsequent localization in these cellular compartments, is critical in performing homeostatic or tumor suppressor functions. Cytoplasmic PTEN suppresses tumors by blocking PI3K/AKT signaling, and nuclear PTEN preserves chromosomal integrity; mislocalization of PTEN in these two compartments may disturb the homeostatic balance, resulting in malignant growth. Several PTEN mutations, including K13E, L320S, and T277A decrease its nuclear accumulation (70). A PTEN Lys289 mutant (K289E) found in Cowden syndrome shows impaired nuclear import but intact catalytic activity (71). Therefore, PTEN subcellular localization is strictly regulated by various regulatory mechanisms.

Phosphatase and tensin homolog may be translocated into the nucleus via multiple channels, including passive transport, monoubiquitination, and SUMOylation of PTEN. Moreover, PTEN import may be mediated by RAS-related nuclear or major vault proteins and putative nuclear localization signal. The size of the nuclear pore is sufficiently large, allowing PTEN protein transport via diffusion. Ras-related nuclear protein GTPase, a 25 kDa protein, is also involved in actively transporting PTEN across the nucleus. The N-terminal of PTEN (residues 19–25) contains a cytoplasmic localization signal. Mutations in these N-terminal residues (except residue 22) of PTEN seem to enhance its nuclear localization (72). The cell cycle stages can also alter the nuclear-cytoplasmic partitioning of PTEN, which then regulates the cell cycle and/or apoptosis status (73).

PTEN post-modification is one of the main mechanisms mediating PTEN translocation. Monoubiquitination of PTEN facilitates its transport across the nuclear membrane (Figure 1). Once in the nucleus, PTEN is deubiquitinated to inhibit the reverse process (71). NEDD4-mediated monoubiquitination at Lys13 or Lys289 PTEN residues promotes its nuclear localization, as evidenced by a substantial expulsion of nuclear PTEN in PTEN K13E-K289E cells, carrying a double mutation of Lysine 289 and 13 residues (71). SUMOylation at the PTEN Lys254 residue in the C-terminal region also promotes its nuclear localization; Lys254-to-Arg mutation eliminated this process without affecting the PTEN catalytic activities in the cytoplasm (74). Herpesvirus- associated ubiquitin-specific protease (HAUSP) counteracts monoubiquitination-mediated PTEN transport (75), while SUMOylation-induced nuclear transfer is reversed by ataxia telangiectasia-mutated (ATM) kinase-mediated phosphorylation (76). Thus, deregulations in PTEN monoubiquitination impairs its nuclear localization, which favors oncogenesis.

Phosphatase and tensin homolog plays numerous critical homeostatic roles in the nucleus, most of which are beyond its lipid phosphatase activity. An in-depth understanding of these dual functions can improve the clinical management of PTEN-deficient human cancers. The absolute loss of PTEN in the cytoplasm promotes cellular senescence and thus cannot be tolerated by glioma cells (18). However, low-grade gliomas progress to advanced grades despite the monoallelic loss or partial loss of PTEN at inception. PTEN dysregulation may occur at the post-translational level or as a result of aberrant subcellular localization in GB (23). Deregulations in the dimer formation of PTEN protein and recruitment to the plasma membrane may also be implicated in its functional loss and tumorigenesis (35). Therefore, PTEN plays an important role in GB, and the mechanism of PTEN needs to be further explored.

## Aberrant Homeostatic Regulation of PTEN in Tumorigenesis

Maintenance of cellular homeostasis depends on the strict control of not only biosynthesis but also the degradation of proteins involved in several signal transduction pathways. Regulation of PTEN activity at post-translational levels is crucial in maintaining PTEN function (25). For instance, ubiquitination plays a dual role in PTEN regulation because it not only participates in the nucleocytoplasmic shuttling and localization of PTEN but also modulates its activity in the cytoplasm (77). Before presenting the details of the ubiquitination-mediated regulation of PTEN proteostasis, other crucial homeostatic mechanisms of PTEN are briefly discussed, including transcriptional and post-transcriptional regulation.

### Transcriptional/Post-transcriptional Regulation of PTEN

Positive transcriptional regulators of PTEN gene expression, including p53, early growth response transcriptional factor 1 (EGR1), activating transcription factor 2, and peroxisome proliferator-activated receptor γ (PPARγ), directly bind to the PTEN promoter (78–80). Negative regulators of PTEN gene expression, including SNAIL and SLUG, compete with p53 to occupy the PTEN promoter (81, 82). PTEN transcription is also negatively regulated by the polycomb complex protein BMI1, c-repeat binding factor 1 (CBF1), c-Jun, and NF-κB (83–85). Encoded by the Notch1 gene, the NOTCH1 protein is a highly conserved cell surface receptor and plays a dual role in PTEN regulation. NOTCH1 promotes PTEN transcription by blocking CBF1/RBPJ and conversely downregulates PTEN by triggering hairy and enhancer of split1 (HES1), an inhibitor (86, 87).

Epigenetic silencing via aberrant hypermethylation of the PTEN promoter causes PTEN gene inactivation in numerous human malignancies, such as breast, melanoma, and lung cancers (88–91). PTEN transcription may also be regulated via histone acetylation. Mechanistically, SALL4 recruits the NuRD complex to the PTEN promoter region that causes histone deacetylation in the region, leading to condensed heterochromatin and repressed PTEN expression (92).

miRNAs (ncRNAs∼14-24 nucleotides), like miR-17, -19, -21, -26, and -214, extensively regulate PTEN at the post-transcriptional level by binding to the response elements of target mRNAs (93). PTEN is also an important target of the miR-17-92 cluster, a polycistronic miRNA cluster, and is overexpressed in lymphoproliferative disorder and autoimmunity (94–96). miR-21 is one of the most frequently upregulated miRNAs in cancer, which targets and inactivates PTEN in multiple human tumors, such as carcinomas of liver, ovarian, and lung origin (97, 98). Similarly, miR-25 negatively regulates PTEN in melanocytes, leading to skin cancer (99–101). miR-214 also promotes the proliferation of tumor cells and induces cisplatin resistance by targeting PTEN in ovarian cancer (102).

### Role of PTM-Ubiquitination in PTEN Regulation

PTEN PTMs play a key role in the indispensable maintenance of diversity and regulate the protein functions of a cell to synchronize their signaling networks. PTMs such as phosphorylation, ubiquitination, SUMOylation, acetylation, and oxidation can dynamically alter the stability, activity, localization, and interaction of PTEN with other proteins.

**FIGURE 2:**
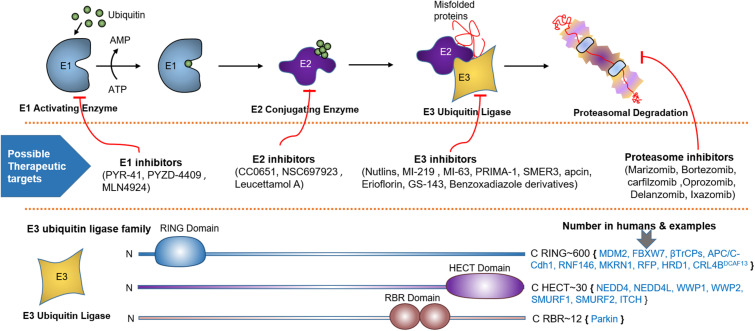
Ubiquitin proteasome system (UPS), spots for therapeutic intervention and PTEN targeting E3 ligases. UPS is a highly sophisticated mechanism that tag proteins for proteasomal degradation through the coordinated actions of a series of enzymes, including E1 activating and E2 conjugating enzymes, and E3 Ubiquitin Ligase. Schematic description shows how E1-E3 enzymes mark substrate protein for degradation by linking it to the ubiquitin polypeptide. Below the UPS pathway, numerous spots for therapeutic intervention in the UPS cascade have been proposed that can either block or enhance ubiquitination. At the bottom, various families of E3 ubiquitin ligase based on unique functional domains, their approximate number in humans and PTEN targeting members are represented.

Ubiquitination involves the covalent attachment of ubiquitin to the PTEN protein that requires several enzymes (71, 103). Ubiquitination of PTEN induced by various E3s, such as NEDD4-1 and WWP2, is implicated in tumorigenesis (104). More than 600 E3s have been identified, which are categorized into three subfamilies based on the mechanism of ligation (Figure 2): HECT, RING-finger (RING), and U-box (105). Ubiquitination affects the subcellular partitioning and degradation of PTEN (71, 103). For instance, single ubiquitination to Lys13 and Lys 289 of the PTEN protein presenting in a loop regulates PTEN nuclear import and stability by monoubiquitination and tumor suppression by polyubiquitination; the loop is formed by the interaction between the C-terminal tail and C2 domains of PTEN (70, 71). Polyubiquitination tags PTEN mainly for proteasomal degradation (70). K48- and K11-linked Ub chains usually cause proteasomal degradation. K63-linked ubiquitination modulates subcellular trafficking/localization, signaling complex assembly, and protein functions. E3s play a crucial role in various cellular processes such as cell proliferation, cell cycle arrest, and apoptosis. Aberrant expression of E3s or mutations in the enzyme is linked to cancer development or suppression (106).

#### RING E3 Ligase Family

The RING E3 ligase family, the largest ligase family, contains important ligases such as the Skp1-Cullin-F-box protein complex (SCF complex) and the anaphase-promoting complex (APC) (107). The RING E3 ligase family plays a role in PTEN homeostasis and thus regulates cancer genesis. RNF146 recognizes ribosylated PTEN, which leads to its ubiquitination and degradation (108). A recent study found that oncogenic MKRN1 is positively regulated via EGF-AKT pathway activation and induces PTEN degradation by ubiquitination (109). Deletion of parkin RBR E3 ubiquitin-protein ligase (PARK2) induces endothelial nitric oxide synthase. This effect improves ROS and Nitric oxide (NO) levels, thus inhibiting PTEN via S-nitrosylation and ubiquitination (110). Ret finger protein (RFP) has been identified as a novel RING E3 ligase that relieves AKT from the negative effect of PTEN via polyubiquitination-induced inhibition of PTEN catalytic activity. However, RFP-mediated polyubiquitination does not affect the stability or localization of PTEN (111). 3-Hydroxy-3-methylglutaryl reductase degradation (HRD1) is a novel E3 ligase that also destroys PTEN by ubiquitination. In hepatocellular carcinoma, negative correlation between HDR1 and PTEN promotes tumorigenesis (112). Human osteosarcoma exhibits CUL4B overexpression, which interacts with DNA damage binding protein 1 (DDB1) as well as DDB1- and CUL4-associated factor 13 (DCAF13). The resulting E3 ligase complex, CRL4B^DCAF13^, leads to the proteasomal degradation of PTEN (113).

#### The Homolog of the E6AP C Terminus (HECT) E3 Ligase Family

This family consists of the HECT domain that is present at the C-terminus of many proteins. HECT E3 ligases regulate diverse physiological pathways, and their aberrant expression marks numerous crucial tumor suppressors for ubiquitin-mediated degradation. The HECT E3 family consists of 28 members, which are further classified into three subgroups; the NEDD4 (nine members) and HERC (six members) subfamilies exhibit similarities in the N-terminal to the HECT domain, whereas the remaining 13 members are designated as “other” HECT E3 ligases. The neural precursor cell-expressed developmentally downregulated 4 (NEDD4) subfamily is the most prominent family and the best characterized, with WW and C2 domains. NEDD4 family members, including NEDD4-1, NEDD4-2 (NEDD4L), ITCH, SMURF1, WWP1, WWP2, NEDL1 (HECW1), and NEDDL2 (HECW2) play key roles in the polyubiquitination-mediated degradation of PTEN in various tumors (Table 1). Nedd4 family-interacting protein 1 (NDFIP1), a PY motif-containing adaptor, recruits the NEDD4 subfamily to PTEN. This recruitment leads to either monoubiquitination-mediated nuclear translocation or polyubiquitination-mediated proteasomal degradation of PTEN (114).

**TABLE 1:** E3 ligases and their PTM mediated effects on PTEN stability and activity.

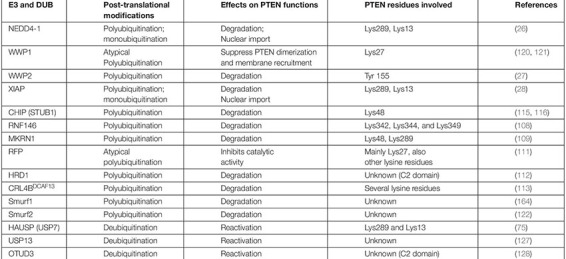

PTEN ubiquitination is regulated by various HECT E3 ligases and chaperone-assisted E3 ligase activity. The X-linked inhibitor of apoptosis protein (XIAP) and its co-chaperone C-terminus of Hsc70-interacting protein (CHIP) play diverse roles in cancers, including glioma where increased expression promotes low- to high-grade transition (28, 115). Overexpression of CHIP in GB increases miR-92b transcription, which reduces PTEN, leading to the hyperactivation of the AKT pathway. PTEN inactivation upregulates AKT, which phosphorylates FOXO1, leading to its ubiquitination and degradation by CHIP (116, 117). Many studies have suggested that CHIP plays tumor suppressor roles, in addition to oncogenic roles. It performs its antitumor functions by regulating oncogenic proteins such as EGFR, a steroid receptor coactivator 3 (SRC3), and androgen receptors in pancreatic, breast, and prostate cancers (106, 118, 119).

WWP1 negatively influences the dimerization and antitumor functions of PTEN via polyubiquitination at K27; depletion of WWP1 raised PTEN protein level in the cells (120, 121). WW domain-containing E3 ubiquitin-protein ligase 2 (WWP2) can more actively tag the unphosphorylated PTEN protein for ubiquitination-dependent degradation (27), compared with NEDD4-1. This difference suggests the importance of phosphorylation profiles for these enzymes (115). SMAD-specific E3 ubiquitin-protein ligase 2 (Smurf2) has recently led to the polyubiquitination and subsequent degradation of endosome-associated PTEN, promoting the activation of non-canonical NF-κB (122). Smurf2 promotes EGFR overexpression-mediated tumorigenesis by protecting EGFR from c-Cbl via its non-proteolytic ubiquitination (123). In breast cancer cells, Smurf2 suppression leads to the downregulation of the PI3K-PTEN-AKT pathway by modulating connector enhancer of kinase suppressor of ras2 (CNKSR2) (124).

NEDD4-1 protein, the most extensively studied E3 enzyme, causes PTEN degradation via polyubiquitination (26). It also facilitates PTEN nuclear import and shuttling between cytoplasmic and nuclear compartments by PTEN monoubiquitination at Lys289 and Lys13 residues (71). This dual-action of NEDD4-1 may be managed by several determinants, including quantity, subcellular localization, regulation via PTMs, and availability of protein adaptors. The role of various E3 adapter (Numb) and activator (NDFIP1 and NDFIP2) proteins in PTEN monoubiquitination or polyubiquitination mediated by NEDD4-1, as well as the underlying mechanisms, has yet to be fully explored (125, 126). Improved insights into physiological signals that regulate the NEDD4-1 switch of PTEN destruction and nuclear translocation will reveal potential therapies.

#### Deubiquitinating Enzymes

Ubiquitin C-terminal hydrolase 13 (USP13), the PTEN deubiquitinating enzyme, has reportedly restored the stability of the PTEN protein in breast cancer cells (127). OUT-domain containing protein 3 (OTUD3) and HAUSP 7 have also been reported for PTEN deubiquitination (75, 128). Guo et al. indicated that Cbl-b E3 protects PTEN from NEDD4-mediated polyubiquitination and degradation in T cells (129). The regulation of PTEN deubiquitination remains poorly understood and requires further investigation. The APC-independent role of CDH1 also negatively regulates WWP2 (130), suggesting that it can protect PTEN and can potentially provide an anticancer strategy (Table 1).

### Role of Other PTMs in PTEN Regulation

#### Phosphorylation

Global mass spectrometry-based studies have revealed 24 phosphorylation sites on PTEN, including C2 domain (Tyr240), phosphatase domain-Tyr46, and the sites located in the serine and threonine residues in the PTEN C-terminal tail (Ser 362, Thr366, Ser380, Thr382, Thr383, Ser370, and Ser385). Phosphorylation of PTEN on tyrosine 240 by FGFR2 mediates resistance to IR therapy and chemotherapy in GB patients. Casein kinase 2 and glycogen synthase kinase 3 phosphorylate Ser370 that mediates the phosphorylation of residues, Thr366, and possibly Ser362 leading to the closed conformation and stability of PTEN (37, 40, 131, 132). Notably, Thr366 seems to slowly auto-dephosphorylate, reducing PTEN localization along the plasma membrane and hence its lipid phosphatase activity (132–135). However, these effects of phosphorylation on the stability of the PTEN protein may vary in different cells. In glioma cells, Thr366 phosphorylation results in protein destabilization (136). The ATM-induced phosphorylation of the PTEN C-terminal tail increases its nuclear translocation (64) and the subsequent induction of autophagy in response to DNA damage (74). Phosphorylation can also modulate the antitumor activity of PTEN. Despite the abundance of the cellular PTEN protein in T cell acute lymphoblastic leukemia (T-ALL), the PI3K-AKT pathway is constitutively upregulated because T-ALL cells block the PTEN function via enhanced phosphorylation (137, 138).

#### SUMOylation

SUMOylation is a ubiquitin-like modification, which involves the attachment of small ubiquitin-related modifiers (SUMOs) to PTEN and other proteins (76). SUMOylation at the Lys266 residue of the PTEN C2 domain promotes its binding to the plasma membrane, leading to PI3K-AKT suppression and cell and tumor growth inhibition *in vivo* (139). SUMOylation at the Lys254 residue also facilitates the nuclear localization of PTEN, promoting the mechanisms of DNA repair (74).

*Other PTMs*, such as oxidation, S-nitrosylation, and acetylation (acetyl group introduction) are also important in PTEN regulation. Reactive oxygen species (ROS) negatively regulate PTEN activity by oxidizing the Cys124 site. Oxidized Cys124 interacts with Cys71 by forming a disulfide bond, which induces a conformational change, thus inactivating the catalytic site of PTEN (138, 140). High intracellular oxidative stress in cancer cells may suppress PTEN, leading to PI3K pathway activation. S-nitrosylation is another redox mechanism that modifies PTEN activity and acts as a therapeutic target (141). Numerous studies have highlighted that NO triggered S-nitrosylation represses the PTEN lipid phosphatase function and promotes the NEDD4-1-mediated polyubiquitination of PTEN (110, 141, 142). Okumura et al. were the first to report on the formation of PTEN catalytic loss pocket caused by acetylation at Lys125 and Lys128 (143). Similarly, PTEN Lys402 acetylation mediated by the p300-CREB-binding protein in the PDZ-binding domain indirectly suppresses PTEN activity by enhancing its protein-protein interactions (144, 145).

## Aberrant Ubiquitination Disrupts Homeostatic Balance of PTEN Protein and Promotes GB Tumorigenesis

The impact of ubiquitination in GB may be cell-context and PTEN mutational status dependent. Importantly, even a subtle reduction in PTEN dosage, such as a hypomorphic allele with 80% of wild-type activity, promotes glioma formation. For example, heterozygous (monoallelic) PTEN loss leads to low-grade gliomas, and also protect cancer cells from immature senescence associated with biallelic PTEN loss. Successive inactivation of PTEN protein, which usually occurs through dysregulated ubiquitination, has dosage-dependent effects on GB progression, latency, and invasiveness. Aberrant expression of several E3 ligases over time dysregulates ubiquitination of PTEN causing additional reductions in PTEN dosage or its mislocalization that leads to accelerated GB progression. Ultimately, complete loss of PTEN protein gives rise to invasive and aggressive GB. Hence, PTEN genetic mutations along with PTM-Ubiquitination could generate a range of aberrant tumor suppressor and oncogene dosages that are advantageous for specific stages of malignant transformation.

**FIGURE 3:**
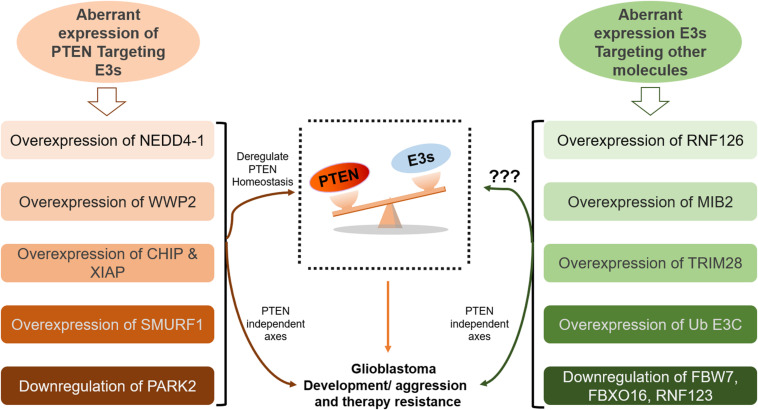
Aberrant homeostasis of PTEN mediated by ubiquitination in GB. Many E3 ligases directly disrupt the homeostatic balance of PTEN tumor suppressor leading to GB development and aggression (on the left side). However, the role of many E3s, which promote GB by deregulating other pathways, in the regulation of PTEN function demand further investigation (on the right side).

Ubiquitination appears to be the major non-genomic mechanism behind PTEN functional loss in several malignancies, including GB. Here, we reviewed several novel E3 ubiquitin ligases that promote GB either by ubiquitination mediated degradation/mislocalization of PTEN or dysregulating other molecular axes (Figure 3). The first reported E3 ligase for PTEN ubiquitination is NEDD4-1 that dysregulates PTEN at the posttranslational level (146). It is the most common E3 ubiquitin ligase that dysregulates PTEN homeostasis in GB. NEDD4-1 mediated monoubiquitination regulates PTEN nuclear shuttling, while polyubiquitination exposes PTEN to proteasomal degradation. Treatment with proteasomal inhibitor (PI) MG132 and shRNA/siRNA mediated suppression of NEDD4-1 raised PTEN protein levels (26). Also, CKI/SCF (β-TRCP) signaling axis negatively regulates NEDD4 and can be targeted to rescue PTEN in human cancers mediated by NEDD4 overexpression (147). Forkhead box protein M1B (FoxM1B) was also found to positively regulate NEDD4-1 expression. Overexpression of FoxM1B in glioma cells and xenograft models upregulated NEDD4-1 that tagged PTEN for proteasomal degradation. Also, overexpression of FoxM1B promoted GB formation in normal human astrocytes (148). A long non-coding RNA, LINC01198, was recently found to enhance ubiquitination mediated degradation of PTEN in glioma. LINC01198 promotes glioma proliferation and confers TMZ resistance by recruiting NEDD4-1 E3 ligase to PTEN (149).

Deletion of the C-terminal region increases PTEN interaction with E3 ligase NEDD4 leading to both mono- and polyubiquitination at K13 and K289 residues on PTEN (71). Yang et al. analyzed PTEN mutations in primary cells derived from 16 GB patients, and reported that some mutations induce defects in PTEN localization at the plasma membrane and/or nucleus. Also, mutation-induced structural abnormalities in the membrane-binding regulatory interface of PTEN open its conformation, hence, exposing it to polyubiquitination mediated degradation. Moreover, an increase in mono-ubiquitination was directly linked with the stability and localization of PTEN (70).

The overexpression of CHIP, an E3 ubiquitin ligase, is also reported in GB that induces ubiquitination mediated removal of PTEN. Knockdown of CHIP suppressed proliferation in U251 and U87 glioma cells, while its overexpression promoted tumorigenesis. *In vivo* studies involving mouse xenograft model recapitulated the results (150). Additionally, activation of CHIP was found to raise the transcription of miR-92b that reduces PTEN in GB (116). WWP2 E3 ligase belongs to the NEDD4-like protein family and has been found to physically interact with PTEN leading to ubiquitination mediated degradation of PTEN (27). WWP2 is abnormally expressed in various tumors including GB and it promotes tumorigenesis mainly by dysregulating PTEN/AKT pathway (151). Overexpression of E3 ligase XIAP is also reported by numerous studies. XIAP was found to regulate PTEN activity and compartmentalization through its mono- and polyubiquitination in HEK-293-T, MCF-7, and HeLa cells (28). Emery IF et al. utilized 50 GB samples and reported that XIAP overexpression was involved in GSCs development and XIAP could be a useful indicator of patient survival (152). Lopez, et al. showed that XIAP knockdown/inhibitors sensitize GB to temozolomide and other chemotherapy drugs (153). Similarly, XIAP inhibitors were shown to enhance the radiosensitivity of glioblastoma cells (154). These studies suggest that XIAP may promote GB genesis through deregulating PTEN protein stability or localization. Deletion of parkin RBR E3 ubiquitin-protein ligase (PARK2) induces endothelial nitric oxide synthase. This effect improves ROS and NO levels, thus inhibiting PTEN via S-nitrosylation and ubiquitination. Gupta et al. reported that PARK2 deficiency hyperactivates PI3K/AKT pathway and this outcome was dependent on PTEN, suggesting that PARK2 loss directly or indirectly impaired the anti-tumor function of PTEN (110).

Overexpression of E3s also promotes GB tumorigenesis by dysregulating several other signaling cascades. RNF126 is overexpressed in glioma tissues, degrading p27, which promotes the growth of glioma cells (155). Loss of p27 or CKI-1B promotes cell cycle progression and glioma cell proliferation, whereas shRNA-mediated silencing of RNF126 reverses these phenotypes. Upregulation of E3 ligase skeletrophin (Mind bomb-2, MIB2) in glioma cells and clinical samples induces resistance to apoptosis via ubiquitination-mediated protection of NF-κB (156, 157). Praja2 deregulates antitumor Hippo cascade in GB by degrading Mps One Binder 1, a positive regulator of this cascade (158). Tripartite motif-28 promotes tumor aggression by negatively regulating p21 (a cyclin-dependent kinase inhibitor) in GB patients (159). Ub E3C (HECT E3 ligase) is overexpressed in GB and marks a tumor suppressor protein, Annexin A7, for ubiquitination and proteasomal destruction; however, Ub E3C exerts no influence on mRNA (129, 160). SMAD Specific E3 Ubiquitin Protein Ligase 1 (SMURF1) also contributes to carcinogenesis via the TGF-β, MAPK, Wnt, and BMP pathways (161, 162). SMURF1 expression is upregulated in GB and mediates metastasis in gliomas by promoting epithelial to mesenchymal translation (EMT), whereas SMURF1 suppression reverses these oncogenic effects (163). Qin et al. also reported upregulated SMURF1 induced ubiquitination mediated degradation of PTEN leading to consistent aberrant activation of PI3K/Akt/mTOR pathway in GB (164). Meanwhile SMURF1 knockdown dramatically inhibits glioma cell proliferation and growth. When combined with mTOR inhibitor rapamycin, SMURF1 silencing resulted in the increased tumor sensitivity to rapamycin leading to significant inhibition of tumor growth in an orthotopic GB model (164). Huwe1 overexpression, observed in multiple organ tumorigenesis, including breast and prostate cancers, tags the tumor suppressor p53 for destruction.

Reduced expression or mislocalization of E3 ligases has also been reported to be oncogenic. Underexpression of PARK2 and the resulting deficiency in its E3 ligase activity frequently occur in GB, leading to mitotic instability and increasing cell proliferation due to unchecked cyclin-E and the Wnt- and EGF-signaling pathways, respectively (165, 166). Dysregulation in β-TrCP1 E3 and subsequent cytosolic β-catenin buildup have been reported in GB. The SCF-E3 enzyme β-transducin repeat-containing protein 1 (SCF^β–TrCP1^) is mislocalized in GB. In normal brain tissues and low-grade gliomas, β-TrCP1 is mainly confined to the cytoplasm, whereas in GB, it is principally transported to the nucleus. This altered localization of β-TrCP1 deregulates its cytoplasmic substrates, including β-catenin and the phosphatase PHLPP (167). FBW7, another E3 ligase of the SCF group, is a critical tumor suppressor. Under hypoxic conditions, FBW7 degrades the antiapoptotic Mcl-1, which is upregulated in GB and promotes Bcl-2 19-KDa interacting protein 3-induced apoptosis (168). Mohsina et al. marked another SCF group E3 enzyme, FBXO16, which functions as a tumor suppressor by the proteasomal degradation of nuclear β-catenin. However, low expression of FBXO16 in GB attenuated its antitumor properties, aggravating Wnt signaling (169). Downregulation of E3-ligase RNF123 was also associated with poor outcomes in GB patients (170). The aberrant buildup of oncoproteins and the degradation of tumor suppressors result in the development and progression of numerous malignancies of GB. Thus, for targeted therapies of GB or other cancers, E3 ligases could be regarded as potential molecules. Moreover, the deubiquitinating enzyme is important for PTEN homeostasis in GB tumorigenesis. The A20 protein has an N-terminal OUT domain, which renders it a deubiquitinating enzyme; the zinc finger domain at its C-terminal confers E3 ligase properties (171, 172). A20, as an E3 ligase, can block TRAIL-induced cell death in GB.

## Development of Oncogenic Addiction to PTEN Loss and Drug Resistance in GB

The absolute loss of the tumor suppressor PTEN promotes cellular senescence leading to the permanent arrest of cell growth/proliferation (19, 173), even in non-proliferating or quiescent cancer stem cells (173, 174); PTEN partial inactivation enhances aberrant growth (16, 18). Thus, GB and other tumors cannot handle the premature complete loss of PTEN activity. Studies found that PTEN-null animals died at the embryonic stage, while PTEN heterozygous mice not only survived but also developed tumors (175, 176). Complete loss of PTEN function is suggested to be a late event in cancer cells that may be induced by the overexpression of certain E3 ligases, leading to the PTEN degradation or dysregulation of its subcellular localization. Overexpression of E3 ligases leads to the loss of PTEN homeostasis; thus, deubiquitinases such as OTUD3 and USP13 stabilize PTEN and restore its homeostatic balance (128). PTEN mutational loss in GB results in the aberrant stimulation of the PI3K-AKT pathway, leading to unchecked abnormal growth and proliferation. Monoallelic loss of PTEN escalates the formation of high-grade astrocytomas (grade III), whereas loss of heterozygosity or biallelic inactivation and AKT activation leads to the development of grade IV tumors (7, 8). These findings suggest that the successive depletion of each PTEN allele is involved in the transition from low- to high-grade gliomas.

Nearly all GBs exhibit PTEN functional loss. Verhaak et al. reported about 85% (145/170 cases of GBs) of the deletion events occur in the PTEN locus, as determined by analysis of The Cancer Genome Atlas data. The frequency of PTEN mutational loss seems comparable in all four GB subtypes, with the classical subtype showing the highest incidence (100%) (6). PTEN monoallelic deleterious mutation is considerably more prevalent in GB (∼30%) than in other major carcinomas (14, 15), disrupting the regulatory and catalytic functions of PTEN (70, 72).

Phosphatase and tensin homolog compete loss mainly occurs in severe subtypes of GB. Yang et al. have recently reported that missense mutations not only deregulate PTEN intracellular localization but also induce protein stability defects. Two missense mutations, PTEN_L320S_ and PTEN_T277A_, induce enzymatically active but functionally inactive PTENs that promote aberrant growth and migration in GB. The C-terminal tail of PTEN generated by these two missense mutations does not relate to the C2 domain at the interface. The absence of association results in abnormally open PTEN conformation and enhanced polyubiquitination-mediated degradation (70, 177). In GB, together with the death-domain associated protein (DAXX) and histone H3 variant (H3.3), PTEN as a component of the chromatin complex also regulates the expression of numerous oncogenes (178). In PTEN-deficient GBs, DAXX terminates the interaction between H3.3 and the chromatin, leading to the chromosomal instability and transcriptional upregulations of oncogenes. Therefore, DAXX inhibition restores H3.3 on the chromatin and limits oncogene expression. DAXX knockdown in PTEN-deficient GB specimens cannot affect the distribution of H3.3, hence the upregulation of tumor suppressors and downregulation of many oncogenes, such as CCND1 and MYC (177). DAXX-mediated heterochromatin maintenance is implicated in telomere stability, and the suppression of pediatric GBs (179).

The frequent occurrence of PTEN mutation in GB has prompted the identification of its antitumor functions (24, 30), further drawing extensive interest in the study of the role of PTEN in deadly malignancies. PTEN loss and hypoxia in astrocytomas enhance the protein expression of the pro-coagulant tissue factor, which promotes thrombosis, angiogenesis, and pseudopalisading necrosis in GB (180). Moreover, in neural stem cells (NSCs), PTEN is usually localized in the nucleus, where it binds to and inhibits the transcription of the paired box 7 (PAX7) promoter. The PTM phosphorylation of nuclear PTEN at the Y-240 residue mediates IR resistance, which promotes GB growth. PTEN deficiency causes PAX7 overexpression, facilitating the carcinogenic transformation of NSCs. Consequently, GB cells become aggressive. Mitomycin C can selectively provoke programmed cell death in PTEN-deficient NSCs (181). Overexpressed SRC and AKT in PTEN-deficient glioma cells induce the activation of the yes-associated protein 1 oncogene and subsequently, lysyl oxidase (LOX). This activation leads to the β1 integrin PYK2-induced enhanced infiltration of tumor-associated macrophages (TAMs) into a GB microenvironment. TAMs promote gliomagenesis by secreting secreted phosphoprotein 1. LOX inhibition via β-aminopropionitrile (BAPN) abates the infiltration of TAMs and tumor growth in PTEN-null animals (182). Moreover, inhibitors of E3s, such as indole-3-carbinol (I3C) for WWP1 and CDH1 for WWP2, can reactivate PTEN. Even monoallelic reactivation is sufficient to antagonize mutation-driven tumorigenesis.

Consistent haploinsufficiency of PTEN and subsequent functional inactivation mediated by E3 overexpression render GB cells adaptive to PTEN loss. Glioma cells seem to have developed oncogenic addiction to the E3–PTEN axis, which promotes their growth and proliferation. This PTEN loss-induced adaptivity confers resistance on EGFR tyrosine kinase inhibitors in GB. Somatic PTEN mutations in GB patients also develop resistance to immune checkpoint inhibitors (Nivolumab: an anti-PD-1 inhibitor) by changing immunosuppressive environments (183). Similarly, mTOR inhibitors activate protein arginine methyltransferase, which provides resistance in mTOR inhibitor therapy in GB (184). Thus, a complete understanding of the genetic status of PTEN in GB, including deletion, inactivating mutation, and post-translational alteration can enhance effective treatments for patients. Inhibitors of oncogenic E3 ligases and other signaling pathways (e.g., the PI3K/AKT/mTOR and Wnt/β-catenin signaling pathways) can be combined to design an effective therapeutic intervention, particularly for the chemotherapy-resistant GB. MLN4924, a NEDD8-activating enzyme inhibitor, blocks ERK and AKT phosphorylation in numerous patient-derived GB stem cells (185). The focal adhesion kinase inhibitor PF-573228 also enhanced p27/CDKN1B levels and β-galactosidase activity and inhibited the autophagy cargo receptor p62/SQSTM-1. This occurrence prompts proliferative arrest and senescence of GB cells (186). Indole-3-carbinol is a potent WWP1 inhibitor that suppresses the PI3K-AKT pathway, leading to PTEN reactivation and tumor suppression (121). PTEN blocks the release of immunosuppressive chemokines by modulating cancer cell secretome, which suppresses the tumor microenvironment by enhancing its immune-permissive characteristic (187). PTEN’s constitutive inactivation leads to multi-organ tumorigenesis, whereas PTEN systemic activation restores the tumor-suppressive state in animals via healthy metabolism. This condition favors the development of treatment modalities that can restore PTEN wt activity (188).

## Conclusion and Perspectives

Glioblastoma is the most deadly brain malignancy, with a median survival rate of up to 1 year after diagnosis. As a potent tumor suppressor, PTEN guards multiple biological processes. These processes include the inhibition of cell growth and proliferation, induction of cell cycle arrest, and promotion of apoptosis. The crucial role of PTEN in cellular homeostasis demands strict regulation of PTEN expression and activity. Loss of PTEN frequently occurs in numerous malignancies, including GB. PTEN loss disrupts the homeostatic balance of biological processes leading to hyperactivation of the pro-survival and oncogenic PI3K-AKT-mTOR pathway. PTEN loss pattern may be unique in different cancers, as excluding glioblastoma and endometrial cancer, PTEN coding sequence mutations are rare. Therapeutic approaches targeting PTEN have not yielded promising outcomes due to multiple functions of PTEN and intricacies in its regulation. Considering the haploinsufficient characteristic of PTEN, we propose that ubiquitination-mediated PTEN loss is more relevant in GB genesis than genetic dysregulation.

PTM-ubiquitination is an indispensable mechanism of proteostasis, and E3 ubiquitin ligases are key members of this process. The impact of ubiquitination in GB may be cell-context and PTEN mutational status dependent. Even a subtle reduction in PTEN dosage, such as a hypomorphic allele with 80% of wild-type activity, promotes glioma formation. Considering the haploinsufficient characteristic of PTEN, an in-depth investigation of PTEN-targeting E3 ligases during GB development can provide a paradigm to explore the molecular mechanisms underlying the dosage-dependent effects of PTEN tumor suppressor and associated oncogenes. Aberrant expression of NEDD4-1 downregulates PTEN function and can potentiate GB tumorigenesis. Moreover, NEDD4-1 can also modulate nuclear PTEN functions as it mediates PTEN nuclear transport via monoubiquitination. Intriguingly, nuclear PTEN is protected from polyubiquitination and subsequent proteasomal degradation by NEDD4-1 (confined mainly in the cytoplasm). Also, NEDD4-1 might promote GB by attenuating the function of heterozygous PTEN more effectively compared to wild type PTEN or its biallelic genetic loss. Hence, understanding of the physiological factors that decide between NEDD4-1 mediated PTEN inactivation versus nuclear translocation could open a new avenue in GB treatment.

Overexpression of CHIP and WWP2 E3 ligases is also implicated in the GB genesis and development and could be a potential biomarker to predict tumor recurrence. Targeting ubiquitin E3 ligases could lead to promising therapeutic interventions to improve the efficacy of chemo- and radiotherapies in GB. Pre-clinical studies involving RNAi mediated silencing of E3s showed that E3 ligases are promising targets for radiosensitization (189). PTEN C-tail phosphorylation could rescue PTEN from HECT E3 ligases (WWP2, WWP1, ITCH, NEDD4-1, etc.) induced ubiquitination and degradation. Of note, interactions between C2/WW domains of HECT E3 ligases and corresponding catalytic HECT domains cause the autoinhibition of these ligases. A peptide linker tethering WW domains can also lock the HECT domain and block the allosteric ubiquitin-binding site leading to the autoinhibition of HECT E3 ligases (190). This shows that E3 ligases are vulnerable and justifies the optimism in developing new therapies targeting this Achilles heel of E3s.

The pro-oncogenic role of various E3s targeting PTEN in GB has been unraveled, however, there is still little known about most of the E3 ligase family members. These E3s include FBXO16, FBW7, β-TrCP1, PARK2, praja2, A20, and SMURF1. E3 ligases are the “brain” of the ubiquitin-proteasome system (UPS), and several small-molecule inhibitors targeting E3s (nutlin and MI-219) have been developed. Also, proteasome inhibitors, PIs (bortezomib, oprozomib, ixazomib, etc.) have shown promising results in clinics but some side effects limit their widespread application (191). Another PI marizomib, which can cross BBB and shows fewer side effects, has been applied to treat newly diagnosed GB (NCT03345095). Advanced technologies, including nucleic acid sequencing, high-throughput and CRISPR screening, and proteomics should be employed to explore more inhibitors for E3 ligases targeting GB. GB, like many other malignancies, has heterogeneity; in addition to aberrant UPS or E3s activity, several other oncogenic signaling cascades may be dysregulated in GB tumorigenesis. This suggests multitarget combination treatment as a future direction.

**FIGURE 4:**
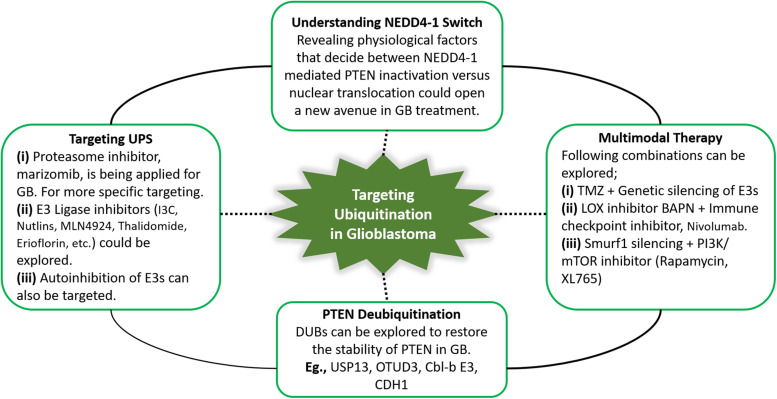
Summary of the potential future directions for targeting PTEN ubiquitination in GB. Therapeutic approaches targeting ubiquitin-proteasome system, specifically ubiquitination can yield promising outcomes by restoring *PTEN* functions. In this context, PTEN deubiquitinating enzymes, and proteasome and E3 ligase inhibitors can be explored in glioblastoma treatment. NEDD4-1 is the most extensively studied E3 ligase. NEDD4-1 causes *PTEN* degradation via polyubiquitination, while regulates *PTEN* nuclear shuttling via monoubiquitination. Improved insights into physiological signals that regulate the NEDD4-1 switch of *PTEN* destruction and nuclear translocation can reveal potential therapies. Keeping in view the heterogeneity in glioblastoma and the involvement of numerous oncogenic players, multitarget combination treatment for glioblastoma should be explored.

Multimodal therapies, including TMZ, mTOR and E3 ligase inhibitors can expand therapeutic interventions for GB patients. In PTEN-null cases, LOX promotes TAM infiltration, leading to immunosuppression in the tumor microenvironment (182). Accordingly, we infer that a T cell checkpoint blockade combined with the LOX inhibitor BAPN may benefit GB patients. Treatment strategies that promote PTEN dimerization also show potential as an anticancer treatment. Furthermore, DUBs (USP13, OTUD3, Cbl-b E3, CDH1, etc.) can be explored to restore the stability of PTEN in GB. Potential future directions for targeting PTEN ubiquitination in GB are summarized in Figure 4. The survival of GB patients can be improved by extensive analysis of PTEN mutations to explore novel targets that may facilitate the development of personalized and targeted therapies to restore the homeostatic balance of the tumor suppressor PTEN.

## Author Contributions

All authors took part in the preparation of this manuscript. QX, SA, and LD were responsible for determining the topic. SA collected the data. QX drew the figures/tables and also contributed with YL and SA. QX wrote the first draft, revised, and edited the manuscript.

## Conflict of Interest

The authors declare that the research was conducted in the absence of any commercial or financial relationships that could be construed as a potential conflict of interest.

## Abbreviations

AKT: protein kinase B
APC/C: anaphase-promoting complex
BAPN: β-aminopropionitrile
CBF1: c-repeat binding factor 1
CDH1: cadherin-1
*CDKN1B*: cyclin dependent kinase inhibitor 1B
CENPC: centromere protein C
CHIP: co-chaperone C-terminus of Hsc70-interacting protein
CNKSR2: connector enhancer of kinase suppressor of ras2
DAXX: death-domain associated protein
DCAF13: DDB1- and CUL4-associated factor 13
DDB1: DNA damage binding protein 1
E3s: ubiquitin ligases
EGFR: epidermal growth factor receptor
EGR1: early growth response transcriptional factor 1
EMT: epithelial to mesenchymal translation
GB: glioblastoma
HECT: homologous to the E6AP C terminus
HES1: hairy and enhancer of split 1
HRD1: 3-Hydroxy-3-methylglutaryl reductase degradation
IR: ionizing radiation
LOX: lysyl oxidase
MCRS1: microspherule protein 1
MES: mesenchymal subtype
mTOR: mammalian target of rapamycin
NDFIP1: Nedd4 family-interacting protein 1
NEDD4: neural precursor cell-expressed developmentally downregulated 4
NSCs: neural stem cells
OTUD3: OUT-domain containing protein 3
PARK2: parkin RBR E3 ubiquitin protein ligase
PBD: PIP_2_-binding domain
PI3K: phosphoinositide 3-kinase
PIP2: phosphatidylinositol 4,5-bisphosphate
PIP3: phosphatidylinositol 3,4,5-triphosphate
PPAR γ: peroxisome proliferator-activated receptor γ
PTEN: phosphatase and tensin homolog
PTMs: post-translational modifications
RFP: Ret finger protein
SCF: Skp1-Cullin-F-box
Smurf2: SMAD-specific E3 ubiquitin protein ligase 2
SQSTM-1: sequestosome 1
SUMOs: small ubiquitin-related modifiers
T-ALL: T cell acute lymphoblastic leukemia
TAMs: tumor-associated macrophages
TMZ: temozolomide
USP13: ubiquitin C-terminal hydrolase 13
WWP2: WW domain-containing E3 ubiquitin protein ligase 2
XIAP: X-linked inhibitor of apoptosis protein.
